# Supplementary material for: Ultra-Low Pt Loading in PtCo Catalysts for the Hydrogen Oxidation Reaction: What Role Do Co Nanoparticles Play?
Source: Nanomaterials (Basel). 2021 Nov 22;11(11):3156. doi: 10.3390/nano11113156 (PMC8625230; doi:10.3390/nano11113156)
Supplement: Supplementary file 1 [file nanomaterials-11-03156-s001.zip › nanomaterials-1398886-supplementary.pdf]

Supplementary Materials

Ultra-Low Pt Loading in PtCo Catalysts for the Hydrogen Oxidation Reaction: What Role Do Co Nanoparticles Play?

Felipe de Jesús Anaya-Castro [1,], Mara Beltrán-Gastélum [1,], Omar Morales Soto[2], Sergio Pérez-Sicairos [1], Shui Wai Lin [1], Balter Trujillo-Navarrete [1,2], Francisco Paraguay-Delgado [3], Luis Javier Salazar-Gastélum [1], Tatiana Romero-Castañón [4], Edgar Reynoso-Soto [1], Rosa María Félix-Navarro [1,†,*] and Moisés Israel Salazar-Gastélum [1,2,†,*]

[1] Tecnológico Nacional de México, Instituto Tecnológico de Tijuana, Centro de Graduados e Investigación en Química, 22510, Tijuana, Mexico; felipe0757@hotmail.com (F.d.J.A.-C.); mara.beltran@tectijuana.edu.mx (M.B.-G.); sperez@tectijuana.mx (S.P.-S.); sl388@aol.com (S.W.L.); balter.trujillo@tectijuana.edu.mx (B.T.-N.); luis.salazarg@tectijuana.edu.mx (L.J.S.-G.); edgar.reynoso@tectijuana.edu.mx (E.R.-S.)

[2] Tecnológico Nacional de México, Instituto Tecnológico de Tijuana, Posgrado en Ciencias de la Ingeniería, 22510, Tijuana, Mexico; omar.soto18@tectijuana.edu.mx

[3] Centro de Investigación en Materiales Avanzados S.C., Laboratorio Nacional de Nanotecnología, 31136, Chihuahua, Mexico; francisco.paraguay@cimav.edu.mx

[4] Instituto Nacional de Electricidad y Energías Limpias, 62490, Cuernavaca, Mexico; tromero@ineel.mx

* Correspondence: rmfelix@tectijuana.mx (R.M.F.-N.); moises.salazar@tectijuana.edu.mx (M.I.S.-G.); Tel.: +52-664-623-3772 (R.M.F.-N.; M.I.S.-G.)

† These authors contributed equally to this work.

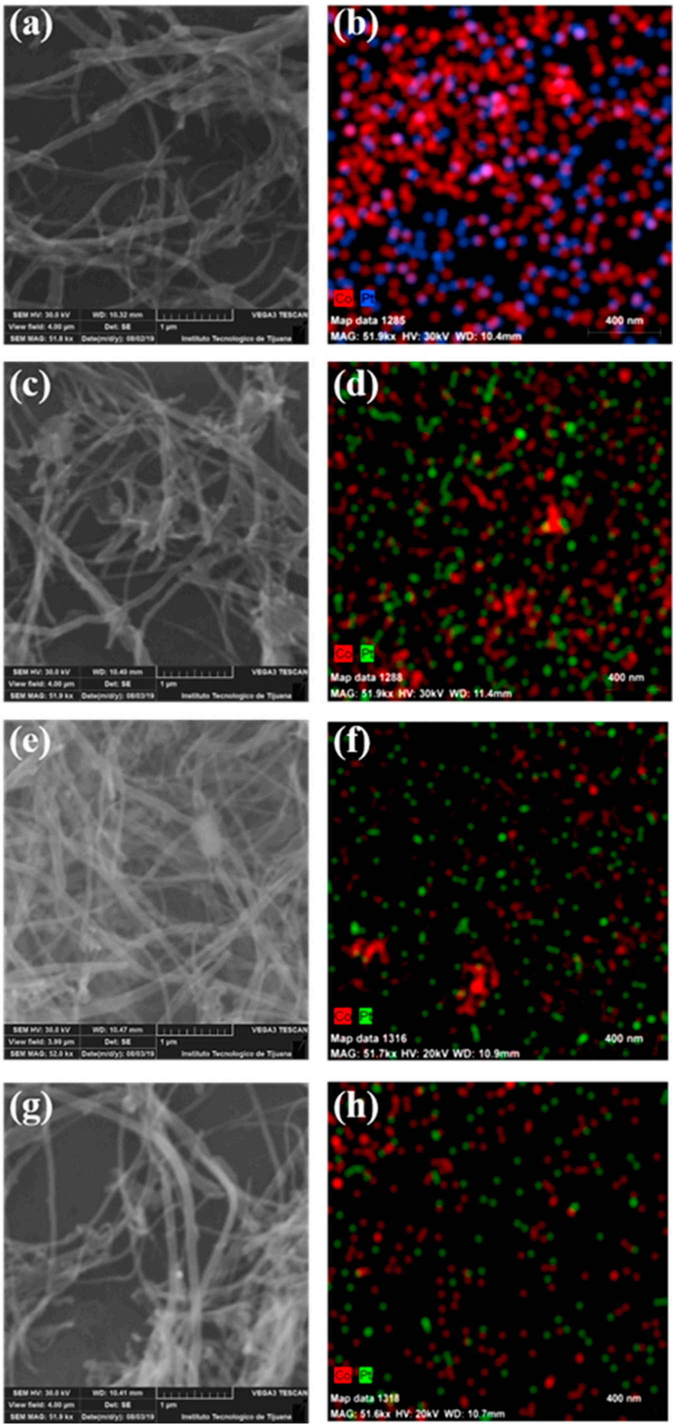

Figure S1. SEM images and EDS mapping analysis of synthesized: PtCo/MWCNT 2 (a-b), PtCo/MWCNT 3 (c-d), PtCo/MWCNT 4 (e-f) and PtCo/MWCNT 6 (g-h).

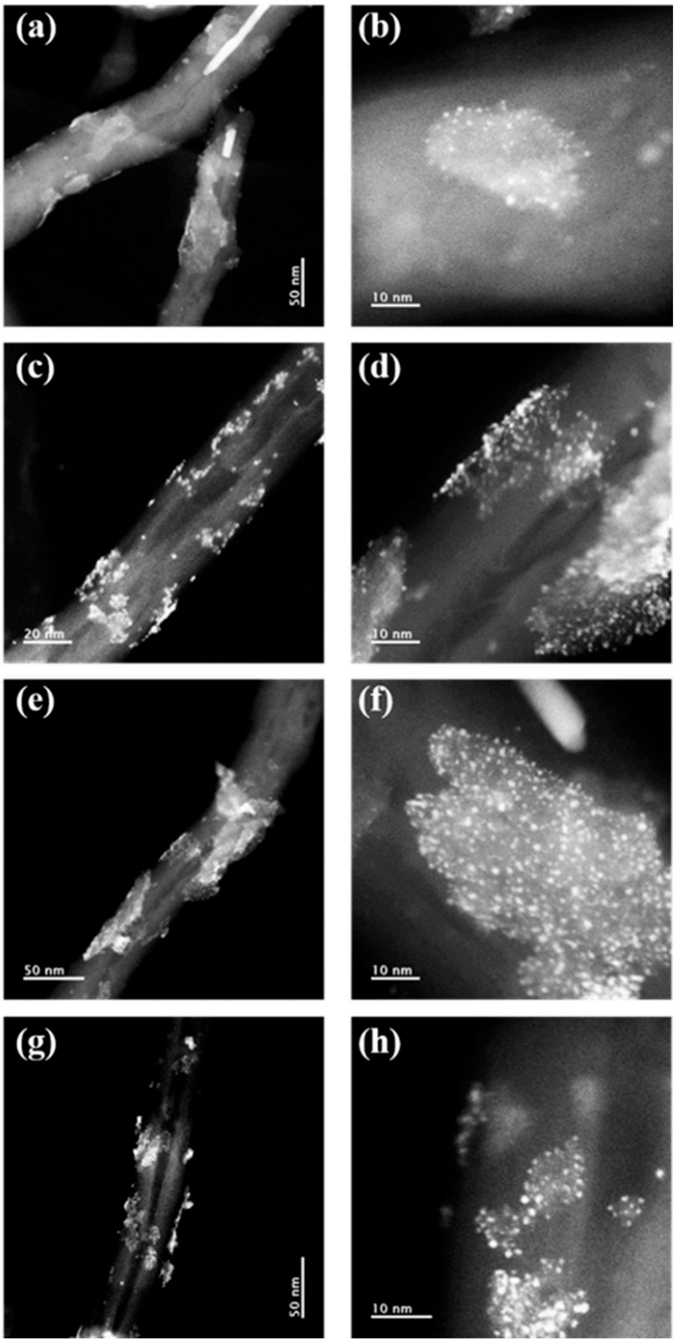

Figure S2. HRTEM images and magnification of Pt NPs on the Co NPs deposited onto MWCNT surface: PtCo/MWCNT 2 (a-b), PtCo/MWCNT 3 (c-d), PtCo/MWCNT 4 (e-f), PtCo/MWCNT 6 (g-h).

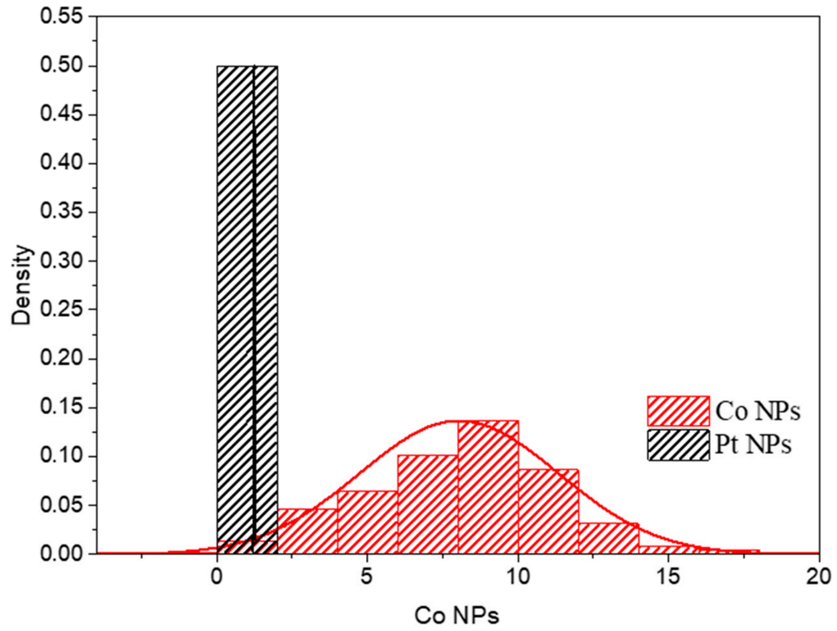

Figure S3. Histogram of Co (red) and Pt (black) NPs deposited on the MWCNT surface for PtCo/MWCNT 4. Both NPs showed probability density curves of the Dp with a Gaussian distribution.
